# Therapeutic Notes

**Published:** 1915-03

**Authors:** J. M. Fortescue-Brickdale


### Therapeutic Notes.

**Camphor** is a drug concerning which there is considerable difference of opinion among clinicians, while experimental pharmacologists also seem uncertain as to the actions which may properly be ascribed to it. In ordinary medicine it is of course largely employed, but so artfully has the official poly-pharmacist combined it, that it is impossible to say to what extent its presence in Tinctura Camphoræ Co. is responsible for the popularity of that time-honoured ingredient in cough mixtures. Externally we are on safer ground, and the irritant and rubefacient action on the skin no doubt contributes to the effect of the compound liniment.

As regards the pharmacology of camphor, some would compare its action to that of picrotoxin, which produces convulsions by stimulating the medulla, while others consider that it resembles cocaine, which in mammals stimulates the cerebral cortex and causes epileptiform fits.

In any case, it appears clear that large doses in mammals ultimately depress the cerebral and medullary centres, causing loss of consciousness and failure of the respiratory centre. Moderately large doses have an action on the heart and respiration, the constancy of which, however, seems doubtful. Camphor is excreted as an inert body combined with glycuronic acid, and it is possible that this combination in some individuals or in certain circumstances may take place so rapidly that the characteristic action of the drug has no time to manifest itself. At any rate, it seems clear that all who profess to get appreciable results in cardiac or respiratory failure from the administration of camphor are unanimous in prescribing large doses.

The British Pharmacopœial dose of camphor is 2 to 5 grains, but it seems clear that larger doses must often be given if any marked action on the central nervous system is to be obtained. The favourite continental method of administration is by means of hypodermic injections of the oil, which is a 20 per cent. solution exactly corresponding to the B. P. liniment. Others prefer a 10 per cent. solution, whilst Viron of the Salpêtrière recommends that 10 per cent. of pure ether should be combined with the same strength of camphor in sterile olive oil purified according to the French codex. Watery 1 per cent. solutions have also been used, but even intravenously they appear to have but little effect. Probably for long-continued administration at least 7 to 10 grains must be given hypodermically in twenty-four hours; good results have been claimed in chronic phthisis by these means. In pneumonia larger doses still have been given—35 grains every twelve hours by some, and 3 grains every three hours by others. One gram to 10 kilograms of body weight, or 150 grains per diem to a man of eleven stone, is recommended by one recent writer.

*Apropos* of these heroic doses, it may not be out of place to remark that the toxic effects of camphor are mental excitement, headache, incoördination, tinnitus, sweating, and occasionally convulsions. Death occurs from respiratory failure.

A single dose of 20–30 grains would probably be followed by toxic symptoms in most adults, and if a series of injections are to be given the effect of any dose above 10 grains should be carefully watched. As it is usually excreted quickly, there would be little danger in repeating a dose found to be innocuous in three or four hours.

Camphor injections have been used to stimulate the heart in poisoning by chloral hydrate and other narcotic drugs; it is stated by some authorities to have a direct action on the cardiac muscle, while Boehm thinks that experimentally the action of camphor is more marked on a heart poisoned by chloral than on a normal organ. Recent experiments have not extended this result to other morbid conditions of the cardiac muscle.

No doubt owing to the doubtful effects of the drug, advocates of its use in pneumonia have suggested that its action may not merely be that of a cardiac or respiratory stimulant, but that it may in some way oppose the pneumococcus or other organism causing the disease. Recent experiments on animals do not appear to give much support to this view. Camphor is of course an antiseptic, and has a general inhibitory action on protoplasm, but this is too weak to have an appreciable effect on a septicæmia in medicinal doses.

**Menthol** is a substance which from the pharmacological point of view is somewhat similar to camphor, though chemically it is not nearly related. Enormous quantities of menthol are prescribed annually in the form of Aqua Menthæ Piperitæ, and externally the drug is not uncommonly used for its local anæsthetic action. But possibly it might with advantage be more frequently prescribed as a serious remedy. Its main uses are as a local anæsthetic and antiseptic, and a vasodilator in diseased conditions of the mucous membranes of the nose and upper respiratory passages and of the stomach and intestines. For coryza it may be employed either as an ointment or dissolved in liquid paraffin (25 per cent.), of which ten drops may be instilled into the nose with a pipette. Olive oil (but not almond oil) is also a suitable vehicle. A teaspoonful of a 5 per cent. alcoholic solution may be used in a steam inhaler, or a similar solution double strength be inhaled from a handkerchief once or twice a day.

For the troublesome headaches which may accompany coryza the following snuff has been recommended :—

Menthol, 1 part.
Boric Acid, 2 parts.
Powdered Orris Root, 4 parts.
Milk Sugar, 4 parts.

In young children and infants severe reflex spasm of the glottis has sometimes been observed when menthol is applied to the nasal mucous membrane, and in adults it has sometimes too irritant an action, causing conjunctivitis, eczema or urticaria.

In the gastro-intestinal tract menthol has been credited with considerable success as a pain-killer. Obviously it will act best where a carminative is required, but its local anæsthetic and antiseptic action may also be valuable. In dyspepsia 2 or 3 grains may be given in a cachet with milk sugar; it is useful in allaying vomiting, provided that no organic lesion such as an ulcer is present in the stomach. It has also been recommended in the vomiting of cholera and dysentery.

As a skin application menthol is mainly used to allay itching, either as an ointment or a spray. For the former it may be combined with equal parts of camphor and chloral-hydrate, and 30 per cent. of the mixture made up with vaseline. As a spray 10 per cent. menthol may be added to equal parts of spirits of camphor, chloroform, and ether.

J. M. Fortescue-Brickdale.

